# Association between the Angiotensin-Converting Enzyme (ACE) Genetic Polymorphism and Diabetic Retinopathy—A Meta-Analysis Comprising 10,168 Subjects

**DOI:** 10.3390/ijerph13111142

**Published:** 2016-11-15

**Authors:** Shasha Luo, Chao Shi, Furu Wang, Zhifeng Wu

**Affiliations:** 1 Department of Ophthalmology, Nanjing Medical University Affiliated Wuxi Second Hospital, 68 Zhongshan Road, Wuxi 214002, China; luoshasha403@163.com; 2 Wuxi Center for Disease Control and Prevention, 499 Jincheng Road, Wuxi 214023, China; shichao.xo@163.com; 3 Jiangsu Provincial Center for Disease Prevention and Control, 172 Jiangsu Road, Nanjing 210029, China

**Keywords:** ACE I/D, polymorphism, diabetic retinopathy, DM type, ethnicity

## Abstract

Aims—to address the inconclusive findings of the association of angiotensin-converting enzyme (ACE) insertion/deletion (I/D) polymorphism on risk of diabetic retinopathy (DR), a meta-analysis was conducted. Methods—we conducted a meta-analysis on 4252 DR cases and 5916 controls from 40 published studies by searching electronic databases and reference lists of relevant articles. A random-effects or fixed-effects model was used to estimate the overall and stratification effect sizes on *ACE* I/D polymorphism on the risk of DR. Results—we found a significant association between the ACE I/D polymorphism and the risk of DR for all genetic model (ID vs. II: OR = 1.14, 95% CI: 1.00–1.30; DD vs. II: OR = 1.38, 95% CI: 1.11–1.71; Allele contrast: OR = 1.17, 95% CI: 1.05–1.30; recessive model: OR = 1.24, 95% CI: 1.02–1.51 and dominant model: OR = 1.21, 95% CI: 1.06–1.38, respectively). In stratified analysis by ethnicity and DM type, we further found that the Asian group with T2DM showed a significant association for all genetic models (ID vs. II: OR = 1.14, 95% CI: 1.01–1.30; DD vs. II: OR = 1.54, 95% CI: 1.14–2.08; Allele contrast: OR = 1.26, 95% CI: 1.09–1.47; recessive model: OR = 1.42, 95% CI: 1.07–1.88 and dominant model: OR = 1.26, 95% CI: 1.07–1.49, respectively). Conclusion—our study suggested that the ACE I/D polymorphism may contribute to DR development, especially in the Asian group with type 2 diabetes mellitus (T2DM). Prospective and more genome-wide association studies (GWAS) are needed to clarify the real role of the ACE gene in determining susceptibility to DR.

## 1. Introduction

Diabetic retinopathy(DR) is the premier cause of vision loss in adults aged 20–74 years [1]. From 1990 to 2010, DR ranked as the fifth most accpeted cause of preventable blindness and moderate to severe visual impairment [2]. Diabetic retinopathy (DR) is a microvascular complication occurring both in type 1 diabetes mellitus (T1DM) and type 2 diabetes mellitus (T2DM), and it was estimated that, of 285 million people worldwide with diabetes, over one-third had signs of DR in 2010 [3]. DR is a complex trait involving polygenic, metabolic, and environmental influences. Known risk factors, most notably the duration of diabetes and glycemic control, explain some, but not all, of the progression of DR [4,5,6]. There are diabetic patients with DR despite short durations of diabetes and/or perfect glycemic control and other diabetic patients who do not develop DR in the face of long-standing diabetes and/or long-term hyperglycemia [7]. Therefore, the genetic factor may explain some of the variation in the progression of DR [8].

The angiotensin-converting enzyme (ACE) gene, plays an critical role in modulating vascular tone through hydrolyzing angiotensin I to vasoconstrictory peptide angiotensin II, which seems to be particularly biologically and clinically relevant to diabetes [9]. A number of studies have reported that patients suffering from DR have high circulating levels of ACE, which implies that elevated serum ACE levels might be a possible hazard factor in destroying retinal vascular apparatus in subjects suffering from diabetes [10]. The ACE gene has a frequent insertion/deletion (I/D) polymorphism characterized by the presence or absence of a 287 bp Alu repetitive sequence in intron 16 [11]. This polymorphism was associated with circulating ACE levels and increased plasma and tissue activity of this enzyme [11,12,13]. Because of the central role of the ACE gene, it is feasible to hypothesize that polymorphism of *ACE* I/D contributes to the development of DR and numerous studies have addressed the role of the variation in the complex etiology of DR.

Numbers of molecular epidemiological studies have been performed to examine the relationship between the *ACE* I/D polymorphism and DR [14,15,16,17,18,19,20,21,22,23,24,25,26,27,28,29,30,31,32,33,34,35,36,37,38,39,40,41,42,43,44,45,46,47,48,49,50,51,52,53,54,55], but the results remain inconclusive. Although several meta-analyses have been published [56,57], they still did not reach a consistent conclusion. To better shed light on these conflicting findings and to quantify the potential between-study heterogeneity and provide better ability to detect smaller effect sizes, we conducted a comprehensive meta-analysis on 40 published studies from 1994 to 2016 with 4252 diabetic retinopathy cases and 5916 controls relating the variant of the *ACE* I/D polymorphism to the risk of developing DR.

## 2. Methods

This study was reported according to the Meta-analysis of Observational Studies in Epidemiology (MOOSE) guidelines and Preferred Reporting Items for Systematic Reviews and Meta-Analyses (PRISMA) for reporting systematic reviews and meta-analyses. Study selection, data extraction, and quality assessment were completed independently by two investigators. Disagreement was resolved through discussion. If the discussion did not lead to a consensus, Professor Wu made the final decision.

### 2.1. Identification and Eligibility of Relevant Studies

All studies that determined the genotype distribution of *ACE* I/D polymorphism in cases with diabetes retinopathy, and (i) in diseased controls (subjects with diabetes and free of DR) or (ii) in healthy controls, were attempted to be included in the meta-analysis. Cases were type 1 or 2 diabetic subjects with background, simple, advanced, or proliferative DR. The control group consisted of two subgroups, the first was the diseased control group, which consisted of subjects with diabetes and which were free of diabetic retinopathy disease, i.e., diabetes nephropathy and myocardial infarct, and the second group was the healthy controls, which was made up of subjects without any diseases.

Studies were firstly identified by searching the electronic literature PubMed for relevant reports in English and CNKI for papers in Chinese (from January 1994 to April 2016, using the search terms “angiotensin converting enzyme” or “ACE” or “rs1799752” in combination with “diabetic retinopathy” or “diabetic retinopathies” or “DR”). We chose articles which were conducted among human subjects. Eligible studies were then identified by further searching the studies published to date on the association between *ACE* I/D polymorphism and diabetic retinopathy risk, and restricted attention to the studies that satisfied all of the following criteria: studies related to the ACE polymorphism were determined regardless of sample size and study design (case-control, cross-sectional, or cohort studies); each genotype frequency was reported, and there was sufficient information for extraction of data; if studies had partly overlapped subjects, only the one with a larger and/or the latest sample size was selected for the analysis. Additional studies were identified by hands-on searches from references of original studies or review articles on this topic. According to these criteria, we finally included 40 papers in our meta-analysis.

### 2.2. Data Extraction and Conversion

Two investigators independently extracted data and reached a consensus on all of the items. Data extracted from these articles included the first author’s name, year of publication, study design, ethnicity of the study population, type of DM, clinical characteristics, and the number of cases and controls for *ACE* I/D genotypes. The frequencies of the alleles and the genotypic distributions were extracted or calculated for both cases and controls. We defined that diabetic patients without retinopathy and/or matched healthy persons constituted the control group, and patients with DR were the case group. We merged the original data into the control group or case group if the study did not provide corresponding data. For some studies without sufficient information for extraction of data, we tried to contact the studies’ authors by sending emails to request data missing from their articles. In addition, it was tested whether the distribution of genotypes in the controls was consistent with the Hardy-Weinberg equilibrium (HWE) for each study, and calculated the frequency of the minor allele for *ACE* I/D polymorphism. 

### 2.3. Quality Assessment and Study Stratification

The Newcastle-Ottawa scale (NOS) method was used to assess the observational included studies. The NOS is composed of three parts (8 entries): selection, comparability, and exposure. A quality item is given only one star for the study in selection and exposure, and a quality item is given, at most, two stars for the study in comparability. It is a semi-quantitative scale, and a score of 0–9 stars is assigned to each study. Studies whose scores were more than 6 stars were considered to be of relatively high quality [58]. The scores of included studies are shown in Table 1.

### 2.4. Meta-Analysis

The meta-analysis evaluated the relationship between the *ACE* I/D polymorphism and the risk of DR for each study by odds ratio (OR), with 95% confidence intervals (95% CI). For all studies, we calculated the ORs for the: (i) separate pairwise comparisons; (ii) allele contrast; (iii) recessive model; and (iv) dominant model. In addition, we conducted stratification analysis by ethnicity and DM type. A sensitivity analysis, which examines the effect of excluding specific studies, was also performed [59]. Our meta-analysis was subjected to sensitivity analysis for studies with the controls not in HWE (*p* < 0.05).

The χ^2^-based Q statistic test was used to assess the heterogeneity, and it was considered significant for *p* < 0.05. Heterogeneity was quantified with the *I*^2^ metric, which is independent of the number of studies in the meta-analysis. *I*^2^ takes values between 0% and 100%, with higher values denoting a greater degree of heterogeneity (*I*^2^ > 50% was considered significant) [60]. We used the fixed-effects model and the random-effects model based on the Mantel-Haenszel method and the DerSimonian and Laird method, respectively, to combine values from each of the studies. When the effects were assumed to be homogenous, the fixed-effects model was then used; otherwise, the random-effects model was more appropriate [61]. In addition, we further conducted meta-regression analyses to estimate the source of heterogeneity. Publication bias was assessed according to the Egger regression asymmetry test and the Begg adjusted rank correlation test [62,63]. All analysis was done by using the Stata software (v.12.1) (StataCorp LP, College Station, TX, USA). All the *p* values were two-sided.

## 3. Results

### 3.1. Literature Search

**Figure 1:**
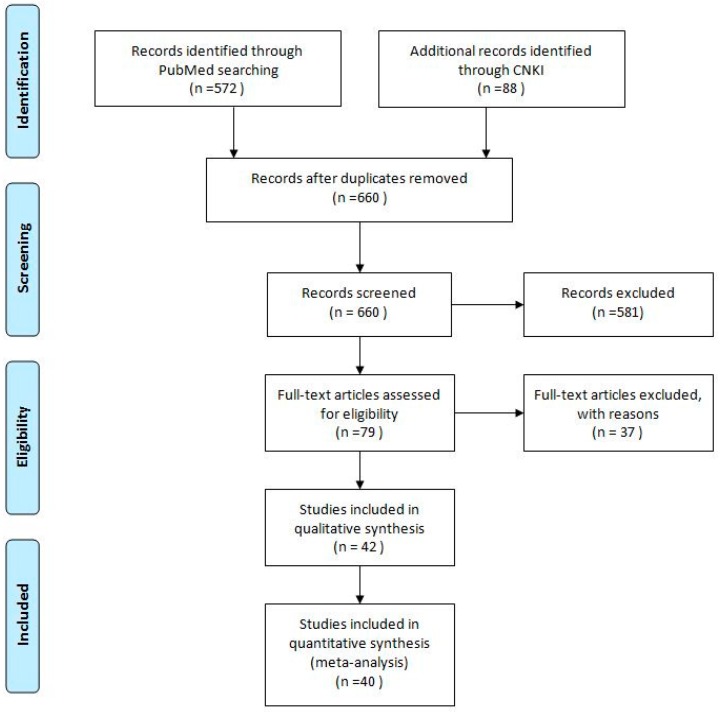
Flow chart of the literature search.

The study selection process is shown in Figure 1. A total of 660 articles (PubMed 572, CNKI 88) were identified from the databases, and 0 duplicates were excluded, using EndNote (X7) (Thomson ResearchSoft, Stamford, CT, USA). In addition, 581 articles were excluded, based on a review of the titles and abstracts, and 79 full-text articles were assessed for eligibility; 37 articles were excluded due to various reasons, such as being review articles or case reports, being written in languages other than English or Chinese, or could not provide each genotype frequency or other sufficient information for extraction of data. Finally, a total of 40 [14,15,16,17,18,19,20,21,22,23,24,25,26,27,28,29,30,31,32,33,34,35,36,37,38,39,40,41,42,43,44,45,46,47,48,49,50,51,54,55] articles were included in this meta-analysis.

### 3.2. Eligible Studies and Study Characteristics

The selected study characteristics from the studies included in the meta-analysis are provided in Table 1, and the details on *ACE* I/D polymorphism allele/genotype prevalence are shown in Table 2. For 40 studies, 8 studies (7 Non-Asian, 1 Asian) involved cases with T1DM, 33 (9 Non-Asian, 24 Asian) with T2DM, and 1 study ([21])with un-defined DM type (1 Asian study with 100 cases and 164 controls). It is worth emphasizing that 2 studies ([17,44]) involved both T1DM and T2DM. The studies on T1DM Non-Asians contributed 599 cases and 614 control subjects, while the Asian studies included 33 cases and 104 control subjects. Among the T2DM studies, studies involving Non-Asians contributed 865 cases and 1541 control subjects, while the Asian studies included 2655 cases and 3659 control subjects. Thirty-three studies were case-control study design, 4 studies were cross-sectional study design, and 3 studies were cohort study design.

### 3.3. Summary Statistics

Data from 40 articles that investigated the association between the ACE I/D polymorphism and DR risk were included in the meta-analysis. The overall frequency (%) of minor D allele frequency (MAF) was 0.47/0.46 for cases and controls. The frequency of the MAF for each study polymorphism on controls is shown in Table 1. All studies suggested that the genotypes distribution in controls was consistent with the Hardy-Weinberg equilibrium except for 8 studies ([22,28,30,35,38,43,48,51]), indicating genotyping errors and/or population stratification [59]; therefore, a sensitivity analysis was performed by excluding these studies.

### 3.4. Main Results, Stratification, and Sensitivity Analyses

**Figure 2:**
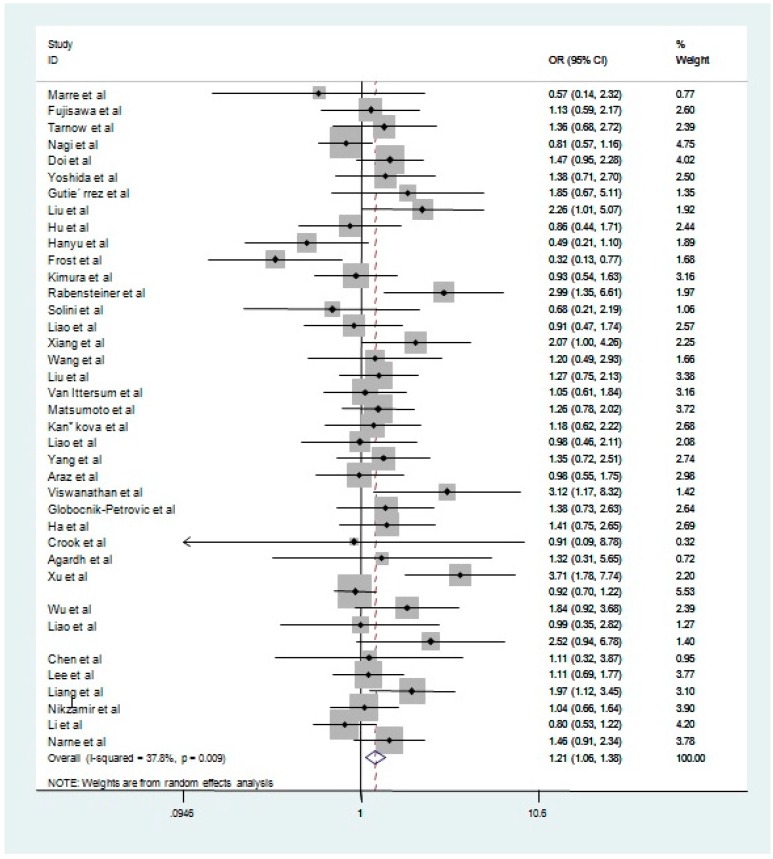
ORs (log scale) of DR associated with *ACE* I/D polymorphism for dominant genetic model. The graph shows individual and pooled estimates for all studies.

The estimation of the relationship of *ACE* I/D polymorphism with DR is presented in Table 3. Figure 2 shows the overall effect for the relationship between the polymorphism and the DR risk in dominant model.

As shown in Table 3, the overall analysis found a significant association between the ACE I/D polymorphism and the risk of DR for all genetic models (ID vs. II: OR = 1.14, 95% CI: 1.00–1.30; DD vs. II: OR = 1.38, 95% CI: 1.11–1.71; Allele contrast: OR = 1.17, 95% CI: 1.05–1.30; recessive model: OR = 1.24, 95% CI: 1.02–1.51 and dominant model: OR = 1.21, 95% CI: 1.06–1.38, respectively).

In a stratified analysis by ethnicity and DM type, we further detected that the Asian group, T2DM group, and Asian group with T2DM all showed significant associations for all genetic models (ID vs. II: OR = 1.14, 95% CI: 1.01–1.29 for the Asian group, OR = 1.13, 95% CI: 1.00–1.24 for the T2DM group and OR = 1.14, 95% CI: 1.01–1.30 for the Asian group with T2DM, respectively; DD vs. II: OR = 1.54, 95% CI: 1.16–2.04 for the Asian group, OR = 1.39, 95% CI: 1.10–1.74 for the T2DM group and OR = 1.54, 95% CI: 1.14–2.08 for the Asian group with T2DM, respectively; Allele contrast: OR = 1.26, 95% CI: 1.10–1.45 for the Asian group, OR = 1.17, 95% CI: 1.04–1.32 for the T2DM group and OR = 1.26, 95% CI: 1.09–1.47 for the Asian group with T2DM, respectively; recessive model: OR = 1.42, 95% CI: 1.08–1.85 for the Asian group, OR = 1.24, 95% CI: 1.01–1.54 for the T2DM group and OR = 1.42, 95% CI: 1.07–1.88 for the Asian group with T2DM, respectively and dominant model: OR = 1.26, 95% CI: 1.08–1.47 for the Asian group, OR = 1.19, 95% CI: 1.05–1.36 for the T2DM group and OR = 1.26, 95% CI: 1.07–1.49 for the Asian group with T2DM, respectively). However, we did not find any significant effects for different genetic models in other subgroup. Further sensitivity analysis for HWE did not alter the pattern of results in both overall analysis and subgroup analysis.

### 3.5. Source of Heterogeneity and Publication Bias

From Table 3, we found that the heterogeneity between studies was observed in overall comparisons as well as subgroup analyses. We estimated the source of heterogeneity in both dominant and recessive genetic models of the variant allele by ethnicity (Asian or Non-Asian), DM type (T1DM or T2DM), HWE (in HWE or not), and study design (case-control, cross-sectional, or cohort study design) by meta-regression analyses. It revealed that none of these four factors could influence significant between-study heterogeneity in genetic models for ACE I/D polymorphism: ethnicity (*p* = 0.78 for dominant model and *p* = 0.39 for recessive model), DM type (*p* = 0.59 for dominant model and *p* = 0.9 for recessive model), HWE (*p* = 0.26 for dominant model and *p* = 0.77 for recessive model), and study design (*p* = 0.06 for dominant model and *p* = 0.24 for recessive model).

**Figure 3:**
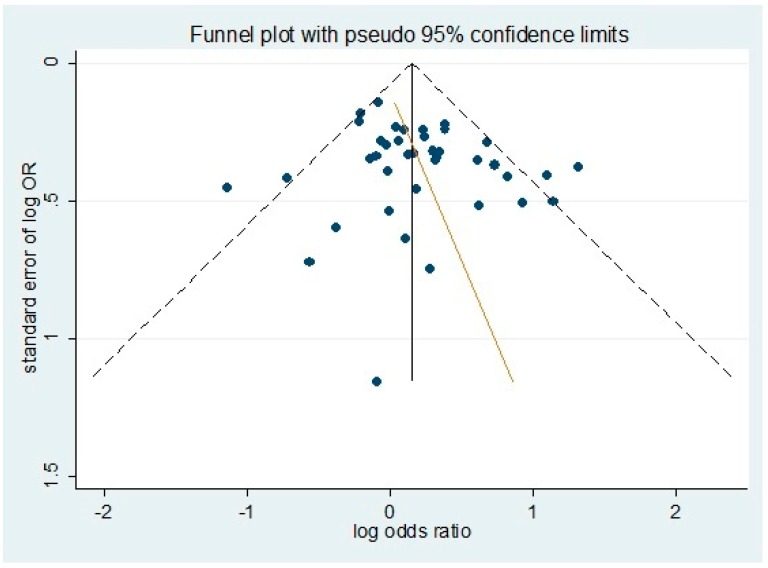
Evaluation of publication bias using funnel plots. Plots are shown for all studies.

The potential presence of publication bias was estimated by using a funnel plot of the evaluation of log-odds ratio for the genotype DD+ ID versus II against the reciprocal of its standard error (Figure 3). As shown, we failed to find any significant funnel asymmetry to indicate publication bias. We further used the Egger regression asymmetry test and the Begg adjusted rank correlation test to estimate the publication bias of literatures included in the meta-analysis. As shown in Table 4, no publication bias was found for polymorphism and risk of DR in genetic models. 

## 4. Discussion

Why some diabetics develop retinopathy, whereas others do not, despite having long-term hyperglycemia, remains an undetermined question. Because known environmental factors do not fully explain this, researchers have sought the answer in the genetic background of the host [32]. The rennin-angiotensin-aldosterone system (RAAS) has been strongly implicated in the pathogenesis of progressive diabetes [64]. The RAAS is a critical regulator of sodium balance, extracellular fluid volume, vascular resistance, and, ultimately, arterial blood pressure by angiotensin II [61,65,66]. Thus, the RAAS serves as one of the most powerful regulators of arterial blood pressure and atherosclerosis and could be considered candidate genes involved in the pathogenesis of diabetic complications, including DR [67,68]. As the gene-encoding components of the RAAS, the ACE gene plays an important role in the RAAS, which is a complicated regulatory network with intrinsic like extrinsic agonistic and antagonistic hormones. It has been increasingly recognized that ACE inhibition demonstrates function and tissue protection of considered organs, to improve eye function of patients with diabetes mellitus and reduce the development and progression of DR [69,70]. In 1990, Rigat et al. described the polymorphism of the ACE gene based on the presence (insertion I) or absence (deletion D) of a 287 base pair element in intron 16 [11]. In plasma ACE levels, this genotype accounts for 47% of the total phenotypic variance in healthy individuals in a way that individuals with D alleles have an increased activity [11]. In addition, Danser et al. showed that the *ACE* I/D polymorphism also influences ACE tissue concentrations [9]. Numerous investigations into the potential role of ACE as a susceptibility gene for DR have been conducted over the past decades, with controversial results. Early meta-analyses attempted to reconcile these findings, but attempts to draw definite conclusions have been hindered by limited data, particularly when examining specific patient subgroups and increased relative studies [56,57].

It is worth emphasizing that our current meta-analysis obtained several critically different conclusions from the previous reports [56,57]. In Zhou’s [56] report, they conducted a separate analysis of only the T2DM and T1DM groups, which showed that the ACE genotype has a non-significant association with DR, regardless of diabetic type. Lu et al. [57] performed the meta-analysis on only the Chinese population, without any subgroup analysis on DM type and ethnicity. However, from the present meta-analysis of 40 studies reported from 1994 to 2016 and comprising 10,168 subjects, we not only found the main effects of *ACE* I/D polymorphism on DR risk, but also found a significant relationship in the T2DM group. From the stratification analysis by ethnicity and DM type, we found that the *ACE* I/D polymorphism was significantly associated with DR risk in the T2DM and Asian groups, especially in the Asian group with T2DM. These findings may indicate that genetic factors may have more impact on the Asian population with T2DM, rather than on other subgroups like the T1DM and Non-Asian population.

We conducted a comprehensive meta-analysis on 40 published studies with 4252 diabetic retinopathy cases and 5916 controls relating the variant of the *ACE* I/D to the risk of DR, which can provide better ability to detect smaller effect sizes. Its strength was based on the accumulation of published data, giving greater information to detect significant differences. In order to estimate the power of the study, we used the Power and Precision 4 software to conduct the power calculation by respectively accumulating the frequency of ACE D allele in case and control groups from all studies, and the result showed the power of our study is 80.2%.

In this study, the effect of separate pairwise comparisons, allele contrast, and the dominant and recessive genetic models were evaluated. Substratification analysis by DM type andethnicity, and sensitivity analysis for studies not in HWE, was performed. In addition, we further evaluated the source of heterogeneity and the publication bias of included literatures.

Despite this, we still have some limits. In the meta-analysis, non-English/Chinese, non-indexed, and non-published studies literature was not reviewed, thus, some bias might be introduced [71]; only the unadjusted pooled ORs were calculated, since data for probable confounding factors that influence the estimates of associations (e.g., age, sex, BMI) were not provided; sampling variability and stratification in genetic association studies could be a possible confounding factor in the role of genetic markers. In addition, the risk effect may depend on the interaction with other risk factors: diabetes duration, HbA_1c_, blood pressure, total serum cholesterol, control of diabetes, and body mass index, all of which modulate the development of DR [3]. Furthermore, small numbers of individuals and inadequate information of lifestyle factors and dietary intake by the published studies limited our statistic power to fully investigate the gene-environment interactions [61]. Therefore, further well-designed large studies, particularly referring to GWAS and gene-environment interactions are warranted to determinate the real contribution of these polymorphisms to DR risk susceptibility and might further indicate the genetics of DR.

## 5. Conclusions

In conclusion, the present meta-analysis finds an association between DR and *ACE* I/D polymorphism, especially in the Asian group with T2DM. Prospective and more genome-wide association studies (GWAS) are needed to clarify the real role of the ACE gene in determining susceptibility to DR.

## Figures and Tables

**Table 1:** Characteristics of published studies included in the meta-analysis.

Author (Reference)	Year	Country	Design	Case				Control				HWE ^#^	MAF *	NOS (Stars *)
				Sample Size	Age (Year)	DM Duration (Year)	Definition	Sample Size	Age (Year)	DM Duration (Year)	Definition			
Marre et al. [14].	1994	France	CC	52	39.0 ± 14.0	20.0 ± 11.0	PDR	32	43.0 ± 18.0	22.0 ± 12.0	IDDM	0.38	0.64	6
Fujisawa et al. [15].	1995	Japan	CC	222	NR	NR	DR	45	NR	NR	NIDDM	0.84	0.36	5
Tarnow et al. [16].	1995	Denmark	CC	155	40.9 ± 9.6	26.7 ± 7.9	PDR	67	42.7 ± 10.2	25.8 ± 8.5	IDDM	0.05	0.57	6
Nagi et al. [17].	1995	Britain	CC	271	50.6 ± 14.3 for IDDM 66.8 ± 10.4 for NIDDM	27 (12–66) for IDDM 11 (1–36) for NIDDM	DR	376	38.3 ± 14.6 for IDDM 69.5 ± 11.1 for NIDDM NA for Healthy	16 (1–56) for IDDM 7 (1–45) for NIDDM NA for Healthy	Healthy + IDDM + NIDDM	0.71	0.52	7
Doi et al. [18].	1995	Japan	CC	362	61.8 (30–79)	>10	DR	105	NA	NA	Healthy	0.25	0.34	4
Yoshida et al. [19].	1996	Japan	CS	118	NA	NA	DR	50	NA	NA	NIDDM	0.59	0.31	4
Gutie’rrez et al. [20].	1997	Spain	CC	68	61.9 ± 9.1	14.8 ± 5.7	DR	92	59.6 ± 10.3	12.1 ± 6.3	NIDDM	0.97	0.61	6
Liu et al. [21].	1997	China	CC	30	NA	NA	DR	198	NA for NDR 34. 8 ± 5. 9 for Healthy	NA	Healthy + NIDDM	0.92	0.27	4
Hu et al. [22].	1998	China	CC	56	62.07 ± 1.21	11.68 ± 0.91	DR	81	56 .06 ± 1 .97 for NDR 56 .86 ± 1 .46 for Healthy	4 .23 ± 0 .47 for NDR	Healthy + NIDDM	0.02	0.35	7
Hanyu et al. [23].	1998	Japan	CC	45	60.0 ± 8.8 without DN 56.1 ± 10.5 with DN	18.2 ± 5.7 without DN 17.0 ± 6.0 with DN	DR	57	56.4 ± 5.1	NR	Healthy	0.72	0.46	6
Frost et al. [24].	1998	Germany	CS	79	30.1 ± 6.6	13.1 ± 8.1	DR	69	30.1 ± 6.6	13.1 ± 8.1	T1DM	0.87	0.67	5
Kimura et al. [25].	1998	Japan	CC	114	NA	NA	PDR	94	43.7 ± 15.4	NR	Healthy	0.14	0.39	6
Rabensteiner et al. [26].	1999	Austria	CC	94	47.2 ± 9.9	31.5 ± 8.2	PDR	81	47.7 ± 11.5	29.7 ± 8.8	T1DM	0.37	0.44	6
Solini et al. [27].	1999	Italy	CS	21	NA	NA	DR	181	NA	NA	T2DM	0.11	0.67	4
Liao et al. [28].	1999	China	CC	68	51.9 ± 11.1 for BDR 53.1 ± 8.8 for PDR	9.35 ± 3.87 for BDR 9.46 ± 5.11 for PDR	BDR+PDR	76	53.2 ± 8.7 for NDR 52.3 ± 9.9 for Healthy	9.29 ± 5.17 for NDR	Healthy + T2DM	0.02	0.37	7
Xiang et al. [29].	1999	China	CC	49	61.1 ± 10.5	7.1 ± 8.2	DR	162	53.2 ± 8.7 for NDR 52.3 ± 9.9 for Healthy	9.29 ± 5.17 for NDR	Healthy + T2DM	0.28	0.38	7
Wang et al. [30].	1999	China	CC	23	58.26 ± 9.57	5.21 ± 5.7	DR	172	59.0 ± 10.0 for NDR 64.9 ± 10.0 for Healthy	4.0 ± 5.1 for NDR	Healthy + T2DM	0.00	0.39	7
Liu et al. [31].	1999	China	CC	100	55 (36–90)	8.8 (0.5–18)	DR	164	53 (38–72) for NDR 35 (20–58) for Healthy	NA	Healthy + DM	0.21	0.40	5
Van Ittersum et al. [32].	2000	New Zealand	CC	101	NA	NA	DR	151	NA	NA	IDDM	0.61	0.46	4
Matsumoto et al. [33].	2000	Japan	CC	120	63.2 ± 10.4 for SDR 56.8 ± 11.9 for ADR	16.7 ± 7.6 for SDR 16.2 ± 9.1 for ADR	SDR+ADR	190	58.9 ± 12.1 for NDR 52.0 ± 1.0 for Healthy	15.0 ± 6.6 for NDR	Healthy + T2DM	0.74	0.38	7
Kankova et al. [34].	2000	Czech	CH	74	NA	NA	PDR	348	63.6 ± 13.4 for Healthy	NA	Healthy + NIDDM	0.19	0.52	5
Liao et al. [35].	2000	China	CC	42	NA	NA	DR	178	54.83 ± 13.71 for NDR 48.71 ± 15.12 for Healthy	0.5–30 for NDR	Healthy + T2DM	0.01	0.54	7
Yang et al. [36].	2000	China	CC	60	NA	NA	DR	137	NA	NA	Healthy + NIDDM	0.21	0.32	4
Araz et al. [37].	2001	Turkey	CS/CC	120	55.0 ± 8.0	11.2 ± 6.5	DR	257	51.0 ± 9.0 for NDR NA for Healthy	5.2 ± 5.1 for NDR	Healthy + T2DM	0.98	0.60	7
Viswanathan et al. [38].	2001	India	CC	86	56.7 + 8.9	13.4 + 6.9	DR	23	56.7 + 9.3	13.2 + 5.1	T2DM	0.01	0.46	6
Petrovic et al. [39].	2003	Slovenia	CC	124	65.6 ± 9.7	18.7 ± 9.1	DR	80	71.3 ± 7.0	16.8 ± 6.8	T2DM	0.07	0.51	6
Ha et al. [40].	2003	Korea	CS	180	NA	NA	DR	59	NA	NA	T2DM	0.07	0.37	4
Crook et al. [41].	2003	USA	CH	46	NA	NA	DR	10	NA	NA	T2DM	0.24	0.80	4
Agardh et al. [42].	2003	USA	CC	24	32 (24–37)	23 (16–31)	SDR	24	28.5 (22–57)	19.5 (10–56)	T1DM	0.74	0.56	6
Xu et al. [43].	2003	China	CC	58	62 ± 10	8 ± 6	DR	142	60 ± 12 for NDR 59 ± 12 for Healthy	8 ± 7 for NDR	Healthy + T2DM	0.03	0.35	7
Thomas et al. [55].	2003	China/Asia	CC	326	59.8 ± 11.4	6.3 (5.6–7.0)	DR	501	60.4 ± 9.3 for T2DM	6.0 (5.6– 6.3)	T2DM	0.38	0.33	6
Wu et al. [44].	2004	China	CH	90	30.5 ± 4.3 for T1DR 60.2 ± 8.3 for T2DR	11.8 ± 2.4 for T1DR 15.1 ± 4.7 for T2DR	DR	294	36.8 ± 6.6 for T1DM 65.2 ± 3.2 for T2DM MI 59.5 ± 1.2 for T2DM NMI	24.3 ± 9.8 for T1DM 15.1 ± 5.0 for T2DM MI 12.3 ± 3.3 for T2DM NMI	T1DM + T2DM	0.22	0.57	8
Liao et al. [45].	2004	China	CC	44	NA	NA	BDR + PDR	21	NA	NA	T2DM	0.16	0.40	4
Degirmenci et al. [46].	2005	Turkey	CC	57	NA	NA	DR	83	NA	NA	T2DM	0.61	0.54	4
Chen et al. [47].	2005	China	CC	27	58.39 ± 9.47	NA	DR	319	55.43 ± 8.31 for NDR NA for Healthy	NA	Healthy + T2DM	0.39	0.63	5
Lee et al. [48].	2006	Korea	CC	130	53.1 ± 12.3	11.4 ± 3.7	DR	174	53.7 ± 12.9	9.4 ± 2.8	T2DM	0.01	0.42	6
Liang et al. [49].	2006	China	CC	82	63.41 ± 11.22	8.34 ± 6.36	DR	153	62.98 ± 11.87 for NDR 65.31 ± 9.77 for Healthy	4.91 ± 4.76 for NDR	Healthy + T2DM	0.54	0.32	7
Nikzamir et al. [50].	2010	Iran	CC	178	59.0 ± 8.7	13 (4–30)	DR	206	59.5 ± 8.2	11 (1–30)	T2DM	0.29	0.46	6
Li et al. [51].	2013	China	CC	207	62.4 ± 7.8	14.6 ± 7.5	DR	302	59.5 ± 8.2 for NDR 75.5 ± 2.8 for Healthy	15.0 ± 4.3 for NDR	Healthy + T2DM	0.02	0.50	7
Narne et al. [54].	2016	India	CC	149	52.7 ± 7.3	14.7 ± 4.7	DR	162	53.4 ± 5.4	15.9 ± 5.6	T2DM	0.05	0.40	6

The reference was referred to the reference numbers in this study; ^**#**^ Hardy-Weinberg equilibrium (HWE) test and * the minor allele frequency (MAF) were calculated in the control group for each study; NR, not reported; NA, not available; CC, case-control; CS, cross-sectional; CH cohort; DR, diabetes retinopathy; BDR, background diabetes retinopathy; SDR, simple diabetes retinopathy; ADR, advanced diabetic retinopathy; PDR, proliferative diabetes retinopathy; NDR, non-diabetes retinopathy; DN, diabetes nephropathy; DM, diabetes mellitus; T1DM, type 1 diabetes mellitus; T2DM, type 2 diabetes mellitus; IDDM, insulin dependent diabetes mellitus; NIDDM, non-insulin dependent diabetes mellitus; MI, myocardial infarct; NMI, non-myocardial infract.

**Table 2:** The details on *ACE* I/D (angiotensin-converting enzyme insertion/deletion) polymorphism allele/genotype prevalence.

Author (Reference)	Prevalence of *ACE* I/D Genotype						Prevalence of Allele Frequency			
	II		ID		DD		I		D	
	Case	Control	Case	Control	Case	Control	Case	Control	Case	Control
Marre et al. [14].	8	3	28	17	16	12	44	23	60	41
Fujisawa et al. [15].	87	19	102	20	33	6	276	58	168	32
Tarnow et al. [16].	29	16	74	25	52	26	132	57	178	77
Nagi et al. [17].	74	88	120	184	77	104	268	360	274	392
Doi et al. [18].	132	48	179	42	51	15	443	138	281	72
Yoshida et al. [19].	45	23	51	23	22	4	141	69	95	31
Gutie‘rrez et al. [20].	6	14	30	44	32	34	42	72	94	112
Liu et al. [21].	10	105	8	78	12	15	28	288	32	108
Hu et al. [22].	29	39	15	27	12	15	73	105	39	57
Hanyu et al. [23].	21	17	18	27	6	13	60	61	30	53
Frost et al. [24].	23	8	25	30	31	31	71	46	87	92
Kimura et al. [25].	48	38	47	38	19	18	143	114	85	74
Rabensteiner et al. [26].	11	23	46	44	37	14	68	90	120	72
Solini et al. [27].	4	25	16	71	1	85	24	121	18	241
Liao et al. [28].	33	35	21	26	14	15	87	96	49	56
Xiang et al. [29].	12	65	23	70	14	27	47	200	51	124
Wang et al. [30].	9	75	8	61	6	36	26	211	20	133
Liu et al. [31].	33	63	38	71	29	30	104	197	96	131
Van Ittersum et al. [32].	29	45	47	72	25	34	105	162	97	140
Matsumoto et al. [33].	41	75	53	87	26	28	135	237	105	143
Kankova et al. [34].	14	75	39	186	21	87	67	336	81	360
Liao et al. [35].	11	46	18	72	13	60	40	164	44	192
Yang et al. [36].	22	60	14	66	24	11	58	186	62	88
Araz et al. [37].	20	42	62	124	38	91	102	208	138	306
Viswanathan et al. [38].	17	10	45	5	24	8	79	25	93	21
Petrovic et al. [39].	28	23	63	32	33	25	119	78	129	82
Ha et al. [40].	48	20	85	34	47	5	181	74	179	44
Crook et al. [41].	5	1	27	2	14	7	37	4	55	16
Agardh et al. [42].	4	5	11	11	9	8	19	21	29	27
Xu et al. [43].	11	66	31	53	16	23	53	185	63	99
Thomas et al. [55].	157	231	129	212	40	58	443	674	209	328
Wu et al. [44].	11	60	45	134	34	100	67	254	113	334
Liao et al. [45].	19	9	16	7	9	5	54	25	34	17
Degirmenci et al. [46].	6	19	34	39	17	25	46	77	68	89
Chen et al. [47].	3	39	5	155	19	125	11	233	43	405
Lee et al. [48].	47	67	69	68	14	39	163	202	97	146
Liang et al. [49].	26	73	36	63	20	17	88	209	76	97
Nikzamir et al. [50].	47	56	73	110	58	40	167	222	189	190
Li et al. [51].	52	64	120	172	35	66	224	300	190	304
Narne et al. [54].	46	64	76	66	27	32	168	194	130	130
**Total**	**1278**	**1854**	**1947**	**2668**	**1027**	**1394**	**4503**	**63,762**	**4001**	**5456**

**Table 3:** Summary ORs and heterogeneity results for associations between the ACE I/D polymorphism and DR (diabetic retinopathy).

Genetic Model	Group	Sensitivity ^#^	Studies	OR	95% CI	*p* *	*I*^2^ (%)
ID vs. II	All studies	All	40	1.14	1.00–1.30	0.02	33.8
		Sensitivity	32	1.08	0.97–1.21	0.13	22.60
	Non-Asian	All	15	1.04	0.86–1.25	0.09	35.30
		Sensitivity	15	1.04	0.86–1.25	0.09	35.30
	Asian	All	25	1.14	1.01–1.29	0.05	34.50
		Sensitivity	17	1.11	0.96–1.29	0.32	11.50
	TIDM	All	8	1.00	0.64–1.56	0.05	50.30
		Sensitivity	8	1.00	0.64–1.56	0.05	50.30
	T2DM	All	33	1.13	1.00–1.24	0.05	31.20
		Sensitivity	26	1.07	1.00–1.21	0.30	11.40
	Non-Asian with T1DM	All	7	0.98	0.84–1.14	0.04	55.40
		Sensitivity	7	0.98	0.84–1.14	0.04	55.40
	Non-Asian with T2DM	All	9	1.03	0.96–1.10	0.49	0.00
		Sensitivity	9	1.03	0.96–1.10	0.49	0.00
	Asian with T1DM	All	1	1.13	0.87–1.46	NA	NA
		Sensitivity	1	1.13	0.87–1.46	NA	NA
	Asian with T2DM	All	24	1.14	1.01–1.30	0.05	36.10
		Sensitivity	16	1.11	1.00–1.29	0.29	13.90
DD vs. II	All studies	All	40	1.38	1.11–1.71	0.00	62.3
		Sensitivity	32	1.46	1.15–1.87	0.00	62.20
	Non-Asian	All	15	1.14	0.81–1.60	0.01	55.50
		Sensitivity	15	1.14	0.81–1.60	0.01	55.50
	Asian	All	25	1.54	1.16–2.04	0.00	65.30
		Sensitivity	17	1.80	1.30–2.51	0.00	63.20
	TIDM	All	8	1.08	0.63–1.87	0.01	61.70
		Sensitivity	8	1.08	0.63–1.87	0.01	61.70
	T2DM	All	33	1.39	1.10–1.74	0.00	61.80
		Sensitivity	26	1.58	1.20–2.07	0.00	66.20
	Non-Asian with T1DM	All	7	1.09	0.92–1.30	0.09	44.90
		Sensitivity	7	1.09	0.92–1.30	0.09	44.90
	Non-Asian with T2DM	All	9	1.06	0.96–1.18	0.26	20.20
		Sensitivity	9	1.06	0.96–1.18	0.26	20.20
	Asian with T1DM	All	1	0.99	0.64–1.53	NA	NA
		Sensitivity	1	0.99	0.64–1.53	NA	NA
	Asian with T2DM	All	24	1.54	1.14–2.08	0.00	66.70
		Sensitivity	16	1.83	1.27–2.63	0.00	65.80
Allele contrast	All studies	All	40	1.17	1.05–1.30	0	64.7
		Sensitivity	32	1.19	1.05–1.35	0.00	65.40
	Non-Asian	All	15	1.02	0.86–1.22	0.00	62.10
		Sensitivity	15	1.02	0.86–1.22	0.00	62.10
	Asian	All	25	1.26	1.10–1.45	0.00	65.40
		Sensitivity	17	1.35	1.15–1.59	0.00	64.00
	TIDM	All	8	1.03	0.78–1.34	0.01	61.00
		Sensitivity	8	1.03	0.78–1.34	0.01	61.00
	T2DM	All	33	1.17	1.04–1.32	0.00	64.90
		Sensitivity	26	1.22	1.06–1.40	0.00	66.50
	Non-Asian with T1DM	All	7	1.02	0.89–1.16	0.01	65.40
		Sensitivity	7	1.02	0.89–1.16	0.01	65.40
	Non-Asian with T2DM	All	9	1.01	0.92–1.10	0.02	54.80
		Sensitivity	9	1.01	0.92–1.10	0.02	54.80
	Asian with T1DM	All	1	0.96	0.76–1.23	NA	NA
		Sensitivity	1	0.96	0.76–1.23	NA	NA
	Asian with T2DM	All	24	1.26	1.09–1.47	0.00	66.90
		Sensitivity	16	1.36	1.14–1.63	0.00	66.30
Recessive model	All studies	All	40	1.24	1.02–1.51	0	67.6
		Sensitivity	32	1.33	1.07–1.66	0.00	69.20
	Non-Asian	All	15	1.03	0.79–1.35	0.00	59.70
		Sensitivity	15	1.03	0.79–1.35	0.00	59.70
	Asian	All	25	1.42	1.08–1.85	0.00	71.10
		Sensitivity	17	1.73	1.24–2.41	0.00	71.90
	TIDM	All	8	1.09	0.86–1.39	0.09	43.20
		Sensitivity	8	1.09	0.86–1.39	0.09	43.20
	T2DM	All	33	1.24	1.01–1.54	0.00	69.50
		Sensitivity	26	1.36	1.06–1.74	0.00	71.90
	Non-Asian with T1DM	All	7	1.09	0.92–1.30	0.09	44.90
		Sensitivity	7	1.09	0.92–1.30	0.09	44.90
	Non-Asian with T2DM	All	9	1.00	0.75–1.25	0.00	67.20
		Sensitivity	9	1.00	0.75–1.25	0.00	67.20
	Asian with T1DM	All	1	0.76	0.42–1.42	NA	NA
		Sensitivity	1	0.76	0.42–1.42	NA	NA
	Asian with T2DM	All	24	1.42	1.07–1.88	0.00	71.80
		Sensitivity	16	1.76	1.23–2.51	0.00	72.90
Dominant model	All studies	All	40	1.21	1.06–1.38	0.01	37.8
		Sensitivity	32	1.17	1.06–1.31	0.05	30.50
	Non-Asian	All	15	1.15	0.97–1.37	0.18	25.30
		Sensitivity	15	1.15	0.97–1.37	0.18	25.30
	Asian	All	25	1.26	1.08–1.47	0.03	37.60
		Sensitivity	17	1.25	1.09–1.42	0.02	19.80
	TIDM	All	8	1.03	0.66–1.61	0.02	57.30
		Sensitivity	8	1.03	0.66–1.61	0.02	57.30
	T2DM	All	33	1.19	1.05–1.36	0.04	32.20
		Sensitivity	26	1.16	1.04–1.29	0.20	18.60
	Non-Asian with T1DM	All	7	1.00	0.90–1.11	0.01	63.00
		Sensitivity	7	1.00	0.90–1.11	0.01	63.00
	Non-Asian with T2DM	All	9	1.02	0.98–1.07	0.67	0.00
		Sensitivity	9	1.02	0.98–1.07	0.67	0.00
	Asian with T1DM	All	1	1.05	0.89–1.25	NA	NA
		Sensitivity	1	1.05	0.89–1.25	NA	NA
	Asian with T2DM	All	24	1.26	1.07–1.49	0.02	40.20
		Sensitivity	16	1.24	1.08–1.43	0.17	25.00

^#^ Sensitivity analysis for HWE; * test for heterogeneity; random-effects model was used when *p* value for heterogeneity test < 0.05 and *I*^2^ > 50%; otherwise, fixed-effects model was used.

**Table 4:** The results of publication bias test by Egger and Begg test.

Sub Group	Egger Test		Begg Test	
	Dominant	Recessive	Dominant	Recessive
all study	0.14	0.71	0.47	0.63
T1DM	0.96	0.86	1.00	1.00
T2DM	0.06	0.62	0.25	0.46
Non-Asian	0.08	0.12	0.11	0.43
Asian	0.09	0.12	0.34	0.18
